# Application of Foley balloon catheter in palliative surgery for pulmonary atresia with an intact ventricular septem, with additional cases of pulmonary atresia with ventricular septal defect and tetralogy of Fallot

**DOI:** 10.1186/s12872-023-03587-z

**Published:** 2023-11-08

**Authors:** Qiteng Xu, Kefeng Hou, Bei Lv, Quansheng Xing, Rui Chen

**Affiliations:** 1 Medical College, Qingdao University, Qingdao, China; 2 Heart Center, Qingdao Women and Children’s hospital, 6 Tongfu Road , Qingdao, 266000 China

**Keywords:** Pulmonary atresia, Tetralogy of Fallot, Palliative surgery, Off-pump, Beating heart

## Abstract

**Background:**

Pulmonary atresia and tetralogy of Fallot can require palliative surgery in the neonatal period due to severe hypoxia; however, limitations of established techniques include high failure rate and need for cardiopulmonary bypass. Herein, right ventricular outflow tract reconstruction on a beating heart using a Foley balloon catheter is described.

**Methods:**

A retrospective review of patients who underwent right ventricular outflow tract reconstruction on a beating heart using a Foley balloon catheter at our institution between September 2018 and March 2022 was completed. During the procedure, a Foley balloon catheter was used to occlude the blood from the right ventricular inflow tract.

**Results:**

Eight patients with pulmonary atresia and intact ventricular septum underwent an off-pump right ventricular outflow tract reconstruction. One patient with pulmonary atresia and ventricular septal defect, and two patients with tetralogy of Fallot underwent an on-pump right ventricular outflow tract reconstruction on a beating heart. The procedures were successful in all patients. Patent ductus arteriosus ligation without modified Blalock-Taussig shunt placement was performed in three patients with pulmonary atresia with intact ventricular septum and two patients with tetralogy of Fallot, ductus arteriosus was left open in four patients with pulmonary atresia with intact ventricular septum. All patients remained clinically well without serious complications.

**Conclusions:**

Right ventricular outflow tract reconstruction on a beating heart using a Foley balloon catheter for pulmonary atresia and tetralogy of Fallot is a feasible alternative to catheter-based interventions or traditional surgical treatment, especially in patients with muscular infundibular stenosis or hypoplastic pulmonary annulus. Further studies with more cases are needed to verify feasibility and superiority of this approach.

## Background

Pulmonary atresia (PA) and tetralogy of Fallot (ToF) are cyanotic congenital heart diseases that can require early establishment of additional pulsatile pulmonary flow, using either surgical or catheter-based interventions, to improve hypoxia and promote growth of the right ventricle (RV) and pulmonary vascular beds [1–3]. To date, several interventions have been employed, such as the Brock procedure under cardiopulmonary bypass (CPB) and modified Blalock-Taussig (mBT) shunt, or catheter-based interventions and hybrid therapy, namely, transthoracic or transventricular balloon pulmonary valvuloplasty (TBPV). However, limitations of these established techniques include need for neonatal CPB, complications due to an mBT shunt, high risk of procedural failure or serious acute complications, and a high neonatal reintervention rate for persistent right ventricle outflow tract (RVOT) obstruction [4–6]. Here, an unique alternative approach for RVOT reconstruction on a beating heart using a Foley balloon catheter for infants with PA and ventricular septal defect (PA-VSD), PA with intact ventricular septum (PA-IVS), or ToF is described.

## Methods

### Patients

This study was approved by the institutional review board of the ethics committee in Qingdao women and children’s hospital (QFELL-YJ-2022-72) on 1 October 2022. Informed consent was obtained in all cases. Between September 2018 and March 2022, a composite group of patients was treated at our institution, comprising 8 cases of PA-IVS, 1 case of PA-VSD(Type A, featuring membranous pulmonary atresia with muscular stenosis of the infundibulum) [7], and 2 cases of TOF. These patients underwent RVOT reconstruction on a beating heart, utilizing a Foley balloon catheter. This approach is contemplated for off-pump RV decompression in patients with PA-IVS characterized by muscular RVOT stenosis, particularly in cases where previous catheter-based interventions for RV decompression proved unsuccessful. Additionally, this technique is applicable in the on-pump Brock procedure in neonates with type A PA-VSD or severe TOF, aiming to circumvent heart standstill.

All patients underwent detailed echocardiographic evaluation, and computed tomography angiography (CTA) was performed before surgery in patients with ToF (*n* = 2) or PA-VSD (*n* = 1). Great attention was paid to tricuspid regurgitation and the Z-value of tricuspid valve(calculated according to the nomogram described by Cantinotti [8]), presence of muscular infundibular stenosis and inner diameter of the patent ductus arteriosus. These details and other patient characteristics are shown in Table 1. In the other three patients with PA-VSD or ToF, various degrees of hypoplasia of the pulmonary artery and its branches were detected. The McGoon ratio and the Nakata index in the PA-VSD and ToF cases, measured from CTA, were 1.14 and 109.23, 1.15 and 55.60, 0.97 and 103.00, respectively. PA-IVS patients who had a successful transcatheter RV decompression, patients with a Z-value of tricuspid valve <-2, patients presented with Ebstein’s malformation or RV-dependent coronary circulation or incomplete three components (the inlet, the trabecular portion, and the outlet) of RV were excluded from this study.

**Table 1:** Patient characteristics

Variables	Value
Diagnosis(PA-IVS/PA-VSD/TOF)	8/1/2
Sex (male/female)	4/7
Mean age at operation (days)	26.2 ± 21.7(5–79)
Mean weight at operation (kg)	3.3 ± 0.5(2.7–4.3)
Tricuspid regurgitation ≥ moderate	8
Mean Z-score of tricuspid valve	-0.06 ± 0.85(-1.18–1.32)
Presence of muscular infundibular stenosis	11
Inner diameter of PDA(mm)	3.5 ± 1.2(2.0–5.4)

*PA-IVS *Pulmonary atresia with intact ventricular septum, *PA-VSD *Pulmonary atresia and ventricular septal defect, *TOF *Tetralogy of Fallot, *PDA *Patent ductus arteriosus

### Procedure

**Fig. 1:**
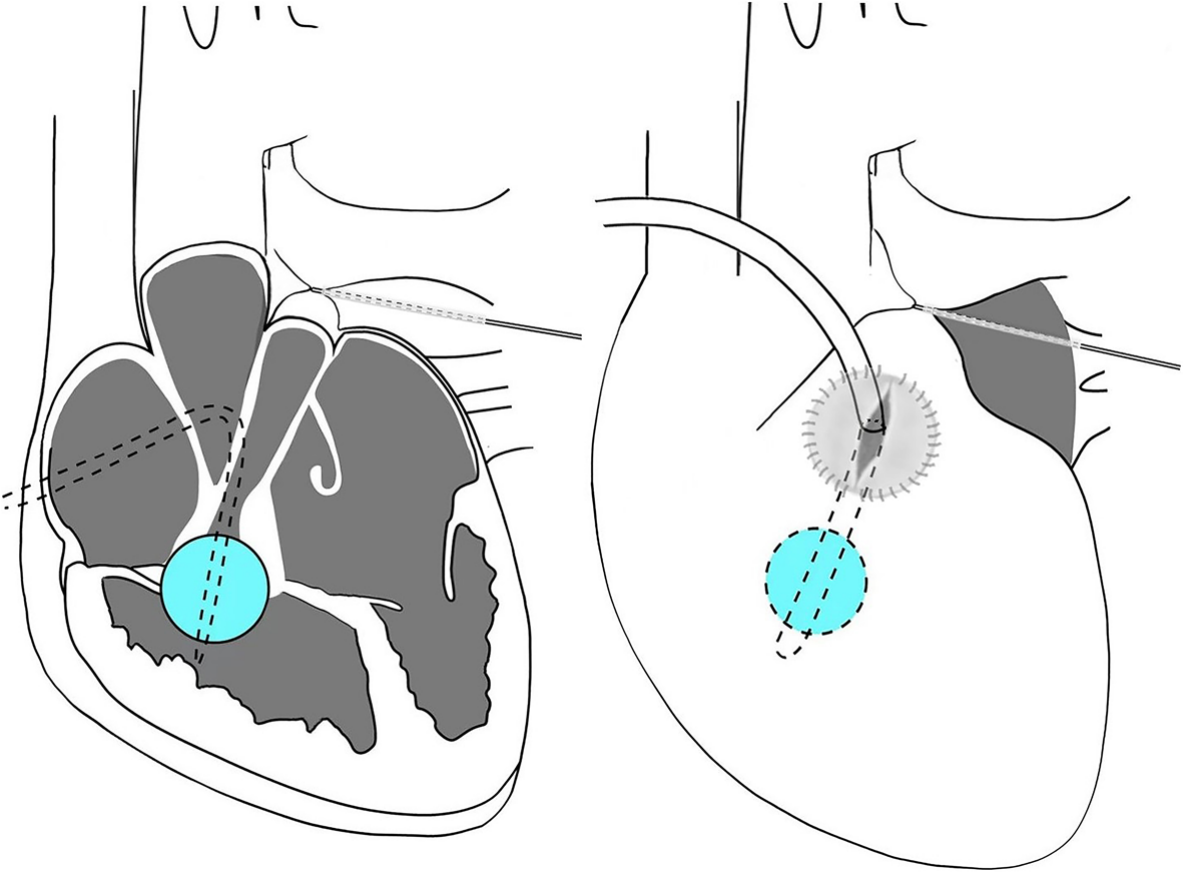
Modified hybrid therapy for PA-IVS, including transventricular pulmonary valvuloplasty and RVOT reconstruction without CPB

All the patients were administered prostaglandin E1(PGE1) to maintain patent ductus arteriosus(PDA) after referral to the intensive care unit (ICU). Eight patients with PA-IVS initially underwent the catheter-based procedure; however, the procedures were unsuccessful despite several attempts to puncture the atresic pulmonary valve. Subsequently, modified hybrid therapy, namely TBPV and RVOT reconstruction without CPB, was performed on these eight patients to depressurize the right ventricle (Fig. 1). Among these cases, two have already been reported by our team with videos [9]. To begin the modified hybrid therapy, circumferential control of the main pulmonary artery (MPA) was obtained after exposing the heart through a median sternotomy in the supine position. Next, a pericardial patch was positioned and sutured over the free wall of the RVOT in advance. After systemic heparinization, traditional hybrid therapy [2, 10, 11] was performed through the incision of the patch. TBPV was then performed under transesophageal echocardiography (TEE) guidance. After final balloon dilation, the catheter was withdrawn and the MPA snare was engaged. Subsequently, a Foley balloon catheter was immediately inserted through the bleeding puncture point such that it headed towards the right ventricular inflow tract, and under TEE guidance, the balloon was first dilated by injecting 2–3ml saline and then clamped to avoid bleeding. Then, dredging of RVOT was performed until two-thirds of the balloon was visible. The Foley balloon catheter was deflated and withdrawn after partial suture of the incision in the pericardial patch. The remaining incision was closed to finish RVOT reconstruction, and a small amount of bleeding during suturing was allowed to avoid air ingression. At the end of the procedure, the MPA snare was released and the PDA was appropriately manoeuvred.

**Fig. 2:**
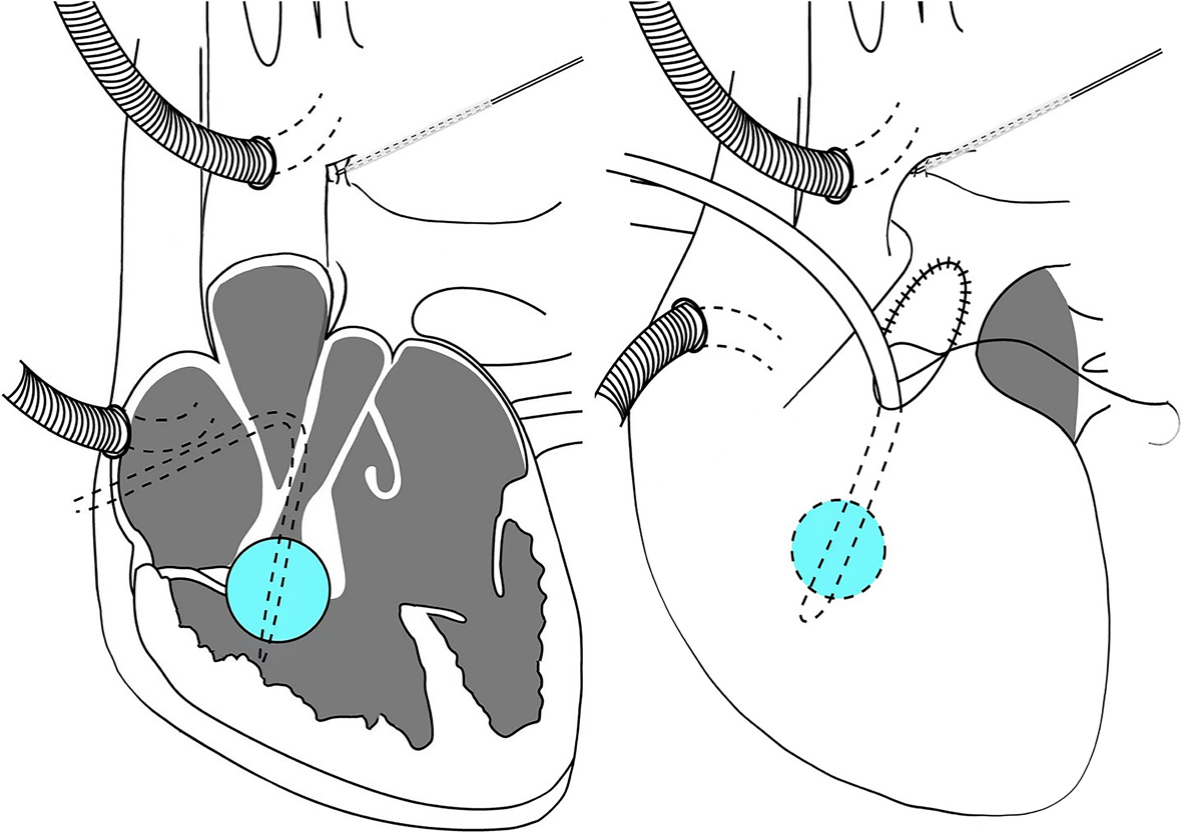
RVOT reconstruction on a beating heart with CPB for PA-VSD (type A, membranous pulmonary atresia with muscular stenosis of infundibulum) and ToF

**Fig. 3:**
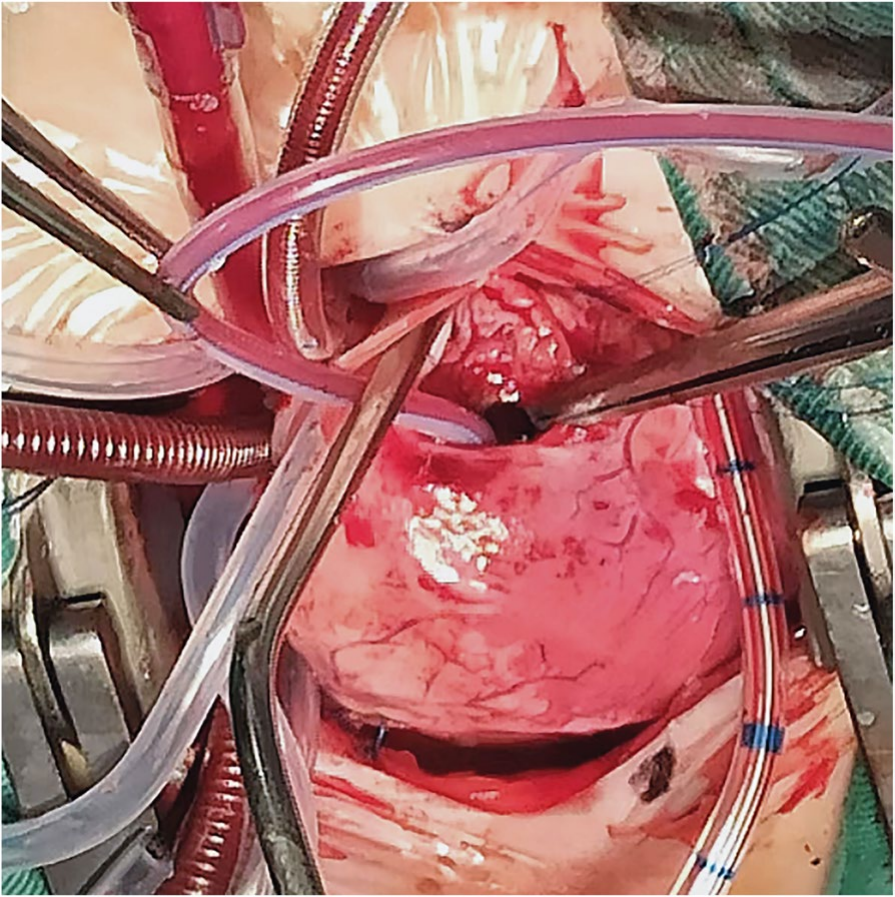
RVOT reconstruction on a beating heart using a Foley balloon catheter

The other three patients with PA-VSD or TOF underwent RVOT reconstruction on a beating heart with CPB (Fig. 2). First, CPB was established and circumferential control of the PDA was obtained. Next, the PDA snare was engaged, a small incision on the free wall of the RVOT was made, and a 6 or 8 F Foley balloon catheter inserted and dilated to occlude blood flow from the right ventricular inflow tract. The procedures used were identical to the ones described above, which resulted in a bloodless operative view and prevented air ingression into the left heart. Next, partial excision of the visible hypertrophic muscles of the RVOT was performed through an extended incision of the RVOT (Fig. 3), and pulmonary valvuloplasty under direct vision and transannular patch augmentation were subsequently performed in two patients(one with PA-VSD and one with TOF) due to the presence of a hypoplastic pulmonary annulus. In contrast, a valve-sparing strategy was adopted in another patient with TOF. The PDA snare was finally released and CPB was stopped.

Except in the first patient with PA-IVS who routinely underwent ligation of the ductus arteriosus and simultaneous mBT shunt placement due to lack of experience, saturation of hemoglobin with oxygen(SpO_2_) was estimated in all the other cases at the end of the procedure by temporarily blocking the PDA. This manoeuvre also helped to decide the strategy of PDA ligation and mBT shunt placement. For patients whose PDA was left open, PGE1 was progressively discontinued after surgery.

## Results

RVOT reconstruction on a beating heart using a Foley balloon catheter was successful in all eleven patients. One patient with PA-IVS and one patient with TOF received the mBT shunt placement. PDA ligation without mBT shunt placement was performed in three patients with PA-IVS and two patients with TOF, and the ductus arteriosus was left open without mBT shunt placement in four patients with PA-IVS.

The median procedure duration was 136.7 ± 41.0 min (range, 96 to 230 min). Median postoperative SpO_2_ with room air increased to 91.2 ± 3.2% (range, 86–96%) from 72.5 ± 9.2% (range, 50–85%). The median mechanical ventilation time was 108.1 ± 82.0 h (range, 23 to 261 h), and median postoperative ICU stay was 12.7 ± 5.2 days (range, 7 to 21 d). The median max velocity in the RVOT was 1.4 ± 0.2 m/s (range, 1.2–1.9 m/s) and median max velocity of pulmonary valve was 2.7 ± 0.7 m/s (range, 1.2–3.6 m/s).

Minor complications occurred in some patients, e.g., PGE1 discontinuation was delayed in one paitent with PA-IVS due to repeated hypoxemia during reduction of pump speed. Nevertheless, PGE1 treatment was finally stopped when a stable SpO_2_ value of 86% with room air, without other interventions, was achieved on day 32 after surgery. Other patients in whom the PDA was left open were successfully weaned from PGE1 in less than one week. Although residual stenosis of the pulmonary valve was common, it was tolerable. Mild pulmonary regurgitation was present in all cases, including in two patients who underwent a transannular patch augmentation procedure.

Patients were followed-up for a median time of 18.6 ± 11.6 months (range, 12–52 months). Out-patient follow-up included echocardiography, electrocardiography, transcutaneous oxygen monitoring, and electroencephalography. A gradual increase in the pressure gradient across the pulmonary valve and consequent hypoxemia were detected in three PA-IVS patients; hence, percutaneous balloon pulmonary valvuloplasty (PBPV) was performed at six months to one year after palliative surgery in them. Unexpectedly, a RVOT aneurysm was detected during PBPV in the first patient with PA-IVS but no apparent worsening of the aneurysm has been observed to date. A closed ductus arteriosus was confirmed by echocardiography at less than one year after the procedure in all patients with PA-IVS. Tolerable maximum pressure gradient at the pulmonary valve, mild regurgitation of the tricuspid valve, and no obvious RVOT obstruction were also detected in all cases. As CTA confirmed well-developed distal pulmonary arteries, definite repair was performed in patients with TOF and PA-VSD at four to twelve months after surgery. Mono-cusp outflow tract patch was used in both cases who underwent the transannular patch augmentation to prevent further pulmonary regurgitation. As CTA revealed local stenosis of right pulmonary artery around the junction with mBT shunt in the patient with PA-VSD, additional patch augmentation of the right pulmonary artery was performed. All patients are doing well clinically without neurological complications.

## Discussion

Neonatal palliative surgery is sometimes essential for relieving cyanosis and for boosting the growth of the native pulmonary arteries in patients with PA or ToF. Different options, typically PBPV, TBPV, mBT shunt, and surgical treatment under CPB, can be considered based on anatomical features and associated anomalies.

PBPV is associated with various drawbacks, including high procedure failure rate and reintervention rate, several complications such as cardiac perforation and early pulmonary regurgitation [2, 5, 6, 12–15]. It is thought that residual infundibular obstruction is responsible for the high reintervention rate [16, 17]. TBPV, despite being associated with fewer intraoperative complications compared to PBPV [2, 10, 11], cannot produce a superior anatomical correction of the infundibular muscular obstruction; hence, muscular stenosis of the RVOT is a proposed contraindication to traditional TBPV [10]. Under most circumstances, mBT shunt placement is needed for the two approaches to improve SpO_2_, which may result in shunt-related complications, such as high risk of occlusion and shunt thrombosis due to the small size of the grafts used, or systemic steal and subsequent heart failure due to overshunting. All of these ultimately result in higher interstage mortality in infants [18, 19]. Additionally, hypoplasia and distortion in the adjoining pulmonary artery have been reported, leading to stenosis or occlusion of the associated pulmonary branches [20].

Theoretically, RVOT reconstruction with transannular patch augmentation or pulmonary valvuloplasty under direct vision yield better anatomical results than catheter-based procedures or traditional hybrid procedures, especially in patients with muscular stenosis of the RVOT or hypoplastic PV annulus. However, the surgical approach requires neonatal CPB, which necessitates a period of cardiac arrest that has both acute consequences related to the systemic inflammatory response and long-term effects on end-organ development and neurocognitive and psychomotor function [21, 22]. Hypothermic cardioplegia also leads to various degrees of cardiac impairment, especially ischaemia and reperfusion injury, leading to postoperative cardiac dysfunction [23].

Thus, a new approach to both the off-pump modified hybrid therapy in PA-IVS and the on-pump RVOT reconstruction with transannular patch augmentation or pulmonary valvuloplasty under direct vision on a beating heart for ToF and PA-VSD (type A, membranous pulmonary atresia with muscular stenosis of infundibulum) is described.

The most critical aspect of this new approach is the use of a Foley balloon catheter to block blood flow from the right ventricular inflow tract. Using 2–3 ml saline to inflate the balloon and achieve appropriate traction of the catheter results in less bleeding and a bloodless operative view, and prevents air ingression into the left heart. This economical new approach may reduce the likelihood of simultaneous mBT placement, decrease the possibility of early reintervention by achieving better anatomical results, and help avoid CPB or cardiac arrest. These aspects would be especially advantageous for patients from low-income countries who suffer from infundibular muscular stenosis or hypoplastic PV annulus.

CPB as a standby for PA-IVS and cerebral oxygenation monitoring are recommended for all. And continuous administration of PGE1 during the procedure are of great importance to ensure adequate supply to the pulmonary artery. Guidance under TEE during the insertion or dilation of the Foley balloon catheter is essential to prevent injuries of tricuspid valve. Mild regurgitation of the tricuspid valve was noted at the latest follow-up for all patients. At the initial stages of a modified hybrid procedure for PA-IVS, a bovine pericardial patch or a glutaraldehyde-treated autogenous pericardial patch previously sutured over the anterior wall of the RVOT is recommended instead of a fresh autogenous pericardial patch because the latter may increase the risk of RVOT aneurysm. An RVOT aneurysm was detected in one patient with PA-IVS in our cohort during catheter reintervention at six months after surgery, which was suspected to be associated with the use of a fresh autogenous pericardial patch. Close follow-up of the RVOT aneurysm continues and further intervention may be inevitable. Therefore, in the subsequent patients, bovine pericardial patches were used as an alternative to cover the anterior wall of the RVOT, and no more RV aneurysms have been detected at one year after surgery. During the RVOT dredging, attentions should be paid to protect the Foley balloon catheter from accidental injury, which is easily avoidable under direct vision. To prevent from pulling out the Foley balloon catheter with excessive resection of the hypertrophic muscles of the RVOT, our experience suggests that it is safe to stop RVOT dredging when about two-thirds of the balloon is visible from the incision.

Further, despite prolonged hospital stays due to sustained PGE1 requirement, it is recommended to avoid mBT placement to prevent the occurrence of associated complications. Hence, our experience was to evaluate the degree of hypoxia during deployment of the PDA snare. Specifically, ligation of PDA without mBT placement was performed if SpO_2_ was higher than 85% during temporary occlusion of the PDA. If SpO_2_ was between 75% and 85%, the PDA was left open without mBT placement. Ligation of PDA with simultaneous mBT placement was performed only if SpO_2_ was lower than 75% during occlusion.

The study presented has a few limitations that should be taken into account. Firstly, the sample size is relatively small, which may limit the generalizability of the findings. Additionally, there is a lack of a concurrent traditional TBPV cohort as a comparison group, which could have provided valuable insights into the effectiveness of the intervention being studied. Furthermore, while all patients were successfully weaned from PGE1, it is important to note that there remains a risk that patients with a reserved PDA may not be able to dissociate from PGE1 and may require further intervention. As such, future studies should aim to address these limitations and explore the potential impact of these factors on the outcomes observed.

## Conclusions

A novel approach to RVOT reconstruction on a beating heart using a Foley balloon catheter in patients with PA-IVS, PA-VSD (type A, membranous pulmonary atresia with muscular stenosis of infundibulum) and ToF is described. This procedure showed encouraging results, is economical, may reduce the likelihood of simultaneous mBT placement, and decrease the possibility of early reintervention due superior anatomical results compared to catheter-based interventions or traditional hybrid therapy. These advantages are especially true in neonatal patients with muscular infundibular stenosis or hypoplastic pulmonary annulus. As our results are limited by small sample size and the retrospective approach, further studies with more cases are needed to verify feasibility and superiority of this novel approach.

## Data Availability

The datasets used and/or analysed during the current study are available from the corresponding author on reasonable request.

## Abbreviations

CPB: Cardiopulmonary bypass
CTA: Computed tomography angiography
ICU: Intensive care unit
mBT: Modified Blalock-Taussig
MPA: Main pulmonary artery
PA: Pulmonary atresia
PA-IVS: Pulmonary atresia with intact ventricular septum
PA-VSD: Pulmonary atresia with ventricular septal defect
PBPV: Percutaneous balloon pulmonary valvuloplasty
PDA: Patent ductus arteriosus
PGE1: Prostaglandin E1
RV: Right ventricle
RVOT: Right ventricle outflow tract
SpO2: Saturation of hemoglobin with oxygen
TBPV: Transthoracic/transventricular balloon pulmonary valvuloplasty
TEE: Transesophageal echocardiography
TOF: Tetralogy of Fallot
